# Intratumoral metastasis of sigmoid colon cancer to chromophobe renal cell carcinoma: a case report

**DOI:** 10.1007/s13691-023-00651-5

**Published:** 2024-02-03

**Authors:** Suzuna Sakai, Kojiro Ohba, Kazunari Migita, Ichiro Sekine, Yasuto Yamazaki

**Affiliations:** 1 Department of Urology, Kouseikai Hospital, Nagasaki, Japan; 2 Department of Urology and Renal Transplantation, Nagasaki University Hospital, Nagasaki, Japan; 3 Department of Surgery, Kouseikai Hospital, Nagasaki, Japan; 4 Department of Pathology, Kouseikai Hospital, Nagasaki, Japan

**Keywords:** Renal cell carcinoma, Metastasis, Colon cancer, Intratumoral metastasis

## Abstract

We herein report an extremely rare case of intratumoral metastasis of colon cancer to chromophobe renal cell carcinoma. A 71-year-old woman was diagnosed with lung metastasis of sigmoid colon cancer and underwent sigmoid colon resection with D3 lymph node dissection. Preoperative contrast-enhanced computed tomography (CT) revealed a left renal tumor; however, colon resection was prioritized, and the renal tumor was placed under observation. Two years later, CT revealed enlargement of the left renal tumor, and laparoscopic partial left nephrectomy was performed 1 month later. Histopathologic examination showed that the resected renal tumor was a chromophobe renal cell carcinoma with intratumoral metastasis of colon cancer to the renal tumor center, and adjuvant chemotherapy with bevacizumab plus SOX (L-OHP + S-1) was initiated. Because of severe chemotherapy-induced fatigue and nausea, the patient was switched to bevacizumab + S-1. However, the patient’s nausea did not improve after this change, and postoperative adjuvant chemotherapy was discontinued at the patient’s request 4 months after the partial nephrectomy. Two months after discontinuation of chemotherapy, CT showed no renal recurrence; however, increased lung metastases and a new bone metastasis in the left sciatic bone were observed. Palliative treatment was then initiated because of severe adverse events that made it difficult to continue treatment. In patients who have multiple cancers and an increase in renal tumor size, the possibility of intratumoral metastasis to the renal tumor should be considered.

## Introduction

Intratumoral metastasis is an uncommon occurrence characterized by the spread of malignant cells from one tumor to within another malignant tumor located outside the primary organ [1]. The kidney is reportedly the most common recipient organ for intratumoral metastasis, and most recipient tumors are clear cell carcinomas; there are few reports of intratumoral metastasis to chromophobe renal cell carcinoma [2, 3]. We herein report a very unusual case of intratumoral metastasis of sigmoid colon cancer to chromophobe renal cell carcinoma.

## Case report

**Fig. 1:**
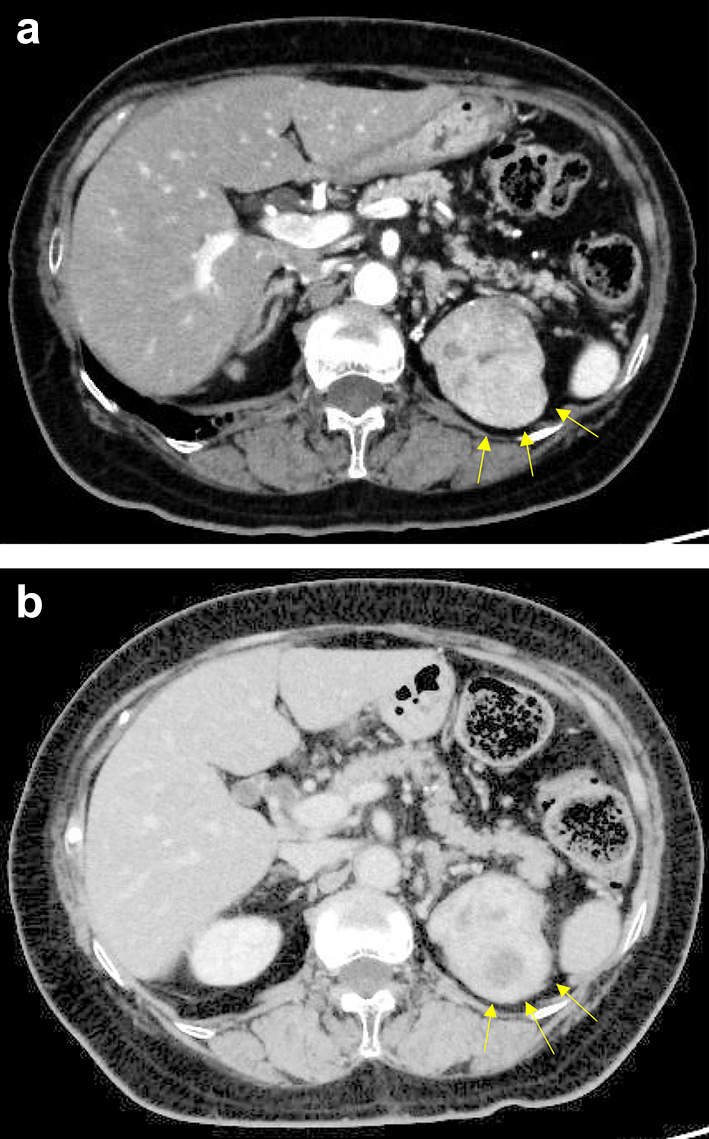
Contrast-enhanced computed tomography image showing a tumor with internal heterogeneous contrast accumulation in the left kidney. **a** Before and **b** after intratumoral metastasis of sigmoid colon cancer to renal cell carcinoma

**Fig. 2:**
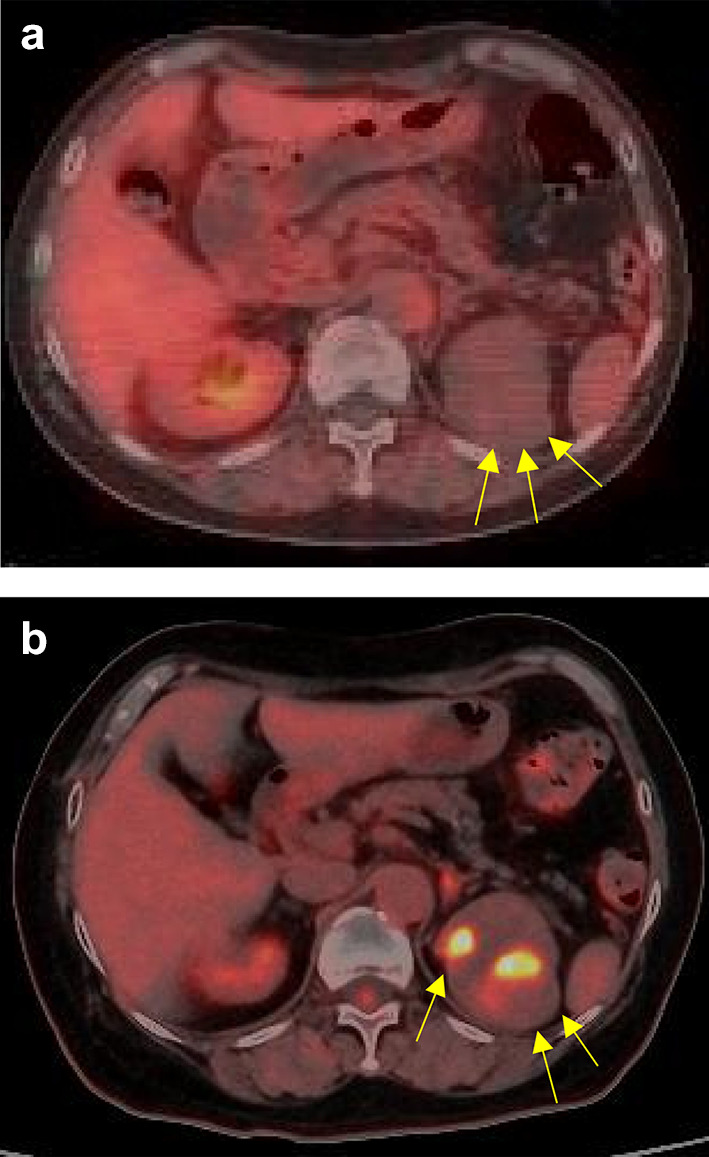
^18^F-fluorodeoxyglucose-positron emission tomography images. **a** No accumulation was present before intratumoral metastasis. **b** Two strong accumulations in the left kidney were observed after intratumoral metastasis

A 71-year-old woman was diagnosed with sigmoid colon cancer with lung metastasis. One month after diagnosis, she underwent sigmoid colon resection with D3 lymph node dissection. Preoperative contrast-enhanced computed tomography (CT) had revealed a tumor in the left kidney, but this tumor was not prioritized for treatment. The definitive diagnosis was sigmoid colon cancer (pT3N0M1), and the patient received chemotherapy with mFOLFOX6 (oxaliplatin [85 mg/m^2^], levofolinate [200 mg/m^2^], and fluorouracil [400 mg/m^2^ bolus followed by 2400 mg/m^2^ over 12 cycles]) plus bevacizumab (Avastin; Genentech, South San Francisco, CA, USA [5 mg/kg]). The patient achieved a partial response with chemotherapy; however, the chemotherapy was discontinued at the patient’s request after two cycles. One year after surgery, she was diagnosed with advanced lung metastasis and underwent robot-assisted right upper lobectomy in another hospital. Contrast-enhanced CT obtained 2 years after the initial surgery revealed that the previously noted left renal tumor had become enlarged to an approximate size of 6 cm, and it exhibited internal heterogeneous contrast accumulation (Fig. 1a, b). ^18^F-fluorodeoxyglucose positron emission tomography (FDG-PET) revealed two sites of strong signal accumulation in the renal tumor (Fig. 2a, b), which differed from the findings observed 1 year earlier. Laboratory tests revealed the following: white blood cell count, 4800/µL (neutrophil count, 2230/µL); hemoglobin, 12.8 g/dL; platelet count, 213,000/µL; creatinine, 0.62 mg/dL; estimated glomerular filtration rate, 71.2 mL/min/1.73 m^2^; calcium, 9.7 mg/dL; C-reactive protein, 0.17 mg/dL; carcinoembryonic antigen (CEA), 366.6 ng/mL (≤ 5.0 ng/mL); and CA19-9, 2.7 U/mL (≤ 37 U/mL). The imaging results and elevated CEA suggested that the left renal tumor was either a primary renal cancer or a left renal metastasis of sigmoid colon cancer. Therefore, the patient underwent laparoscopic partial left nephrectomy 1 month later.

The patient was placed under general anesthesia in the right lateral recumbent position, and a transabdominal approach was used. A camera port was placed on the outer edge of the rectus abdominis muscle two finger widths cephalad to the umbilicus, and a 12-mm port was placed 7 cm cephalad to the camera port. After dissection of the adhesions between the abdominal wall and mesentery, a 12-mm port was placed 7 cm lateral to the camera port, and an assistant 12-mm port was placed caudal to the camera port. The tumor was located in the suprarenal pole lateral to the kidney, and tumor resection was performed from the ventral to dorsal side. The operative duration was 4 h 30 min, and the pneumoperitoneum time was 3 h 42 min. The total blood loss was 50 mL, the ischemia time was 18 min, and the excised tumor was 75 × 65 × 40 mm in size.

**Fig. 3:**
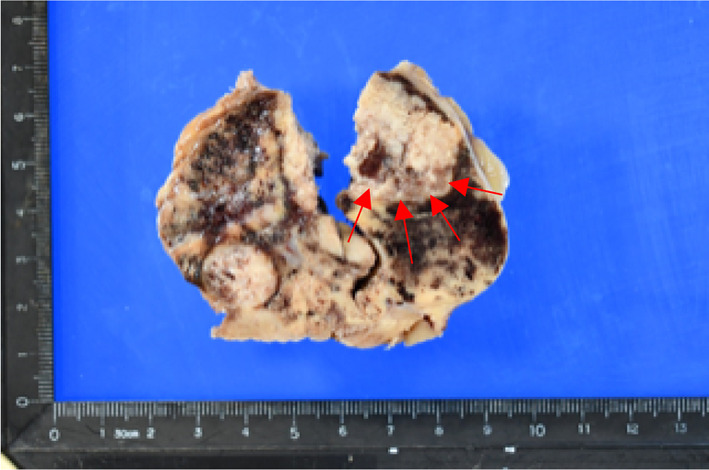
Gross appearance of the resected renal tumor. Arrows indicate colon cancer metastases

**Fig. 4:**
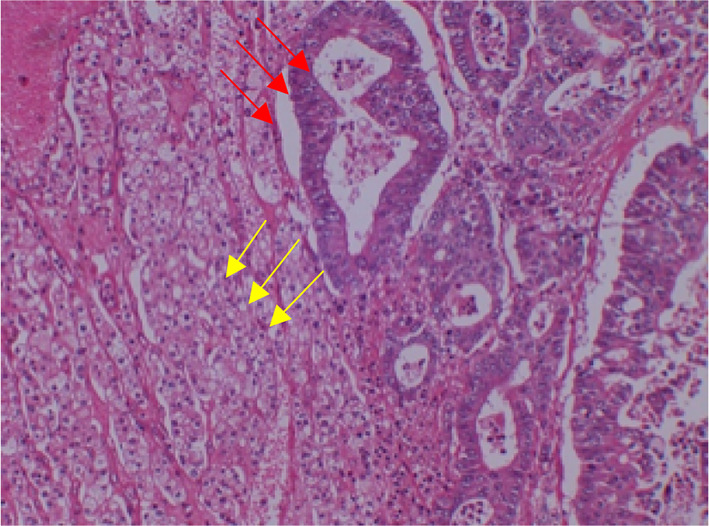
Hematoxylin/eosin staining of chromophobe renal cell carcinoma (yellow arrows) with intratumoral metastatic carcinoma (red arrows). Magnification, 100 ×

**Fig. 5:**
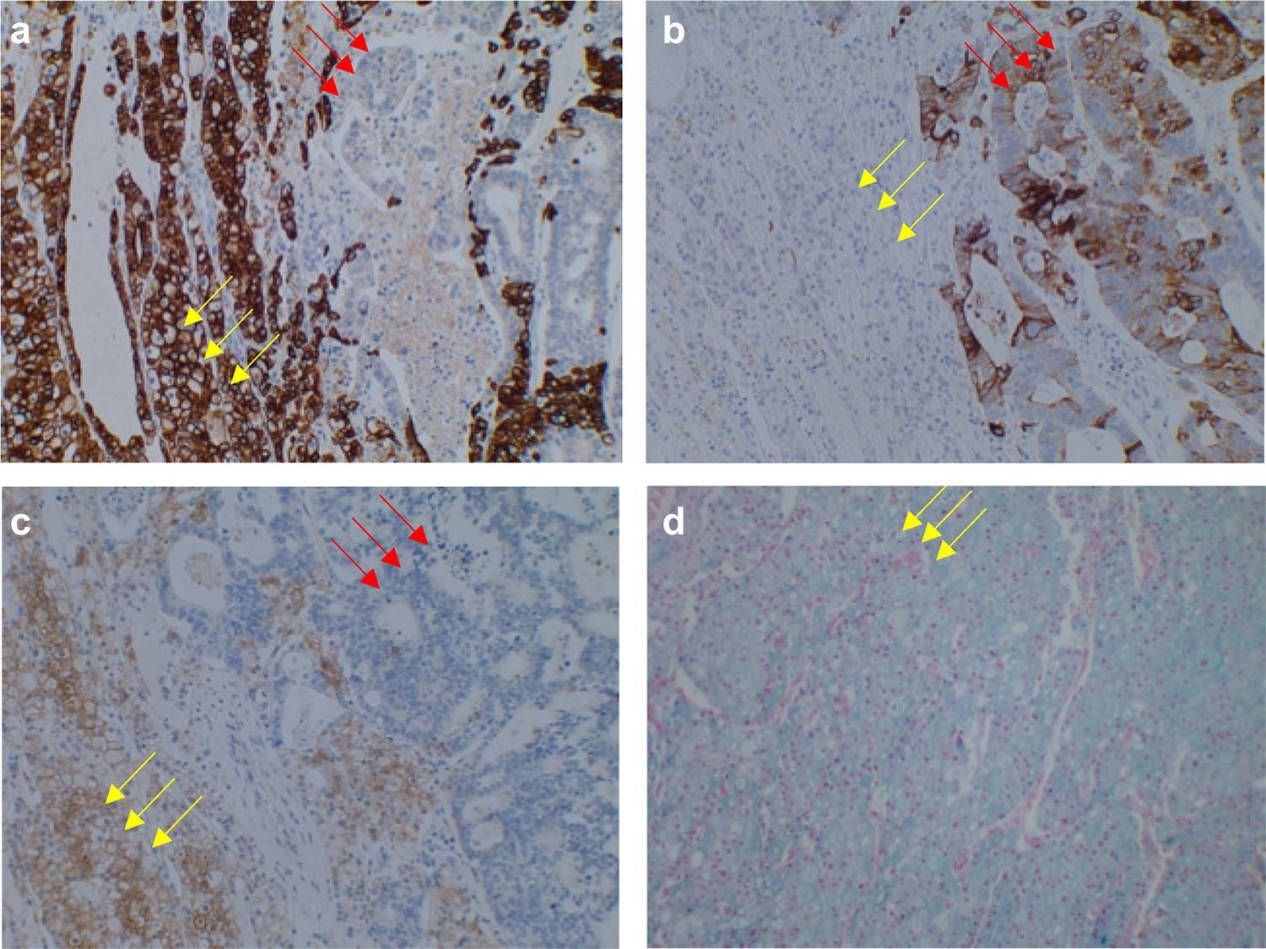
Immunostaining of chromophobe renal cell carcinoma (yellow arrows) with intratumoral metastatic carcinoma (red arrows). **a** CK7. **b** CK20. **c** c-kit. **d** Colloidal iron. Magnification of all panels, 100 ×

Histopathologic examination revealed that the tumor was a chromophobe renal cell carcinoma (expansive type, 70 mm in maximum tumor diameter, Grade 2; WHO/ISUP grading system atypia classification, Grade 2; Fuhrman classification, LyX, VX, pT2a, INFa, eg, fc1, imX, rc-inf0, rp- inf0, s-inf0) (Fig. 3). In addition, multiple colon cancer metastatic lesions were observed inside the renal tumor, including a mass measuring 30 mm. Histologic examination of the intratumoral metastatic lesions revealed highly to moderately differentiated tubular adenocarcinoma (Fig. 4). Immunostaining (Fig. 5) revealed AE1/AE3 positivity in the renal carcinoma, colon carcinoma, and normal renal tubules; CK7 positivity in the renal carcinoma and normal renal tubules; CK20 positivity only in the colon carcinoma; and c-kit and colloidal iron positivity in the chromophobe renal cell carcinoma. By contrast, CA9 immunostaining was negative throughout. Based on these results, the definitive diagnosis of the renal tumor was chromophobe renal cell carcinoma. The central portion of the tumor was consistent with the histologic features of the previously resected sigmoid colon cancer: this region was CK7-negative and CK20-positive by immunostaining, confirming the diagnosis of intratumoral metastasis of sigmoid colon cancer to chromophobe renal cell carcinoma.

Postoperative laboratory tests revealed the following: creatinine, 0.70 mg/dL; estimated glomerular filtration rate, 62.3 mL/min/1.73 m^2^; and CEA, 40.4 ng/mL. Multiple lung metastases were observed 2 months after the partial nephrectomy (PN), and chemotherapy with bevacizumab plus SOX (capecitabine + oxaliplatin [130 mg/m^2^] + S-1 [100 mg]) was initiated for the lung metastasis of colon cancer. CT showed no renal recurrence, but multiple lung metastases and a new bone metastasis in the left ischium were observed. The patient declined further treatment, and chemotherapy was, therefore, discontinued. Six months after PN, brain metastasis appeared. Radiotherapy for the brain metastasis was being performed at the time of this writing.

## Discussion

Metastases to the kidney, which are rarely detected in living patients, are typically from primary tumors originating in the lungs (35.1%–46.3%) and uterus (11.8%–27.0%); metastasis from the colon is less common (2.9%–4.0%) [4]. Some reports have proposed four mechanisms regarding the metastasis of colon cancer to the kidneys: hematogenous metastasis via the lungs, transcortical hematogenous metastasis from the liver via the lungs, retrograde metastasis with direct invasion of the ureter, and implantation of tumor cells to retroperitoneal tissue at the time of initial surgery [5, 6]. Given the presence of pulmonary metastasis in the present case, the metastasis was considered to have occurred through hematogenous spread to the kidney.

Intratumoral metastasis is defined as the presence of two or more tumors, where the metastatic site itself is a true tumor and the metastatic tumor is confirmed as a true malignant tumor in vivo. Metastasis to a lymphoreticular tumor is excluded from this definition [7, 8]. Furthermore, the metastases are definitely intratumoral and not collision carcinomas, the primary tumor is definitely present, and the two tumors are morphologically and immunohistochemically different. Intratumoral metastasis generally occurs in high-grade tumors, and the metastatic sites are often indolent tumors [9]. Ueno et al. [3] reported that renal carcinoma was a prevalent recipient tumor in cases of intratumor metastatic lesions. Several potential mechanisms should be considered for this observed prevalence. First, there is a rich blood flow to the kidneys. Second, renal cancer and the donor tumor can be easily differentiated by immunohistochemistry. Third, the stroma of renal cancer is rich in capillaries, fat, and glycogen, and contains little connective tissue, providing an ideal environment for the metastasis and growth of another tumor type. Clear cell renal carcinoma, with its abundant blood flow, is by far the most common histologic recipient tumor type in the kidneys. Reports of metastasis to chromophobe renal cell carcinoma with poor blood flow, such as that described herein, are extremely rare [3, 10].

Cancer metastasis is assumed to have organ selectivity or specificity, and the two known mechanisms of metastasis are the anatomical-mechanical theory and the seed and soil theory [11, 12]. Tumor metastasis requires both the ability of tumor cells to form metastases and the participation of host-related factors. A recent report indicated that the expression of chemokine receptors is involved in tumor metastasis [13]; thus, the involvement of some chemokines may induce intratumoral metastasis. Various factors such as the expression of adhesion molecules, proteolytic enzymes, and cell migration-promoting factors may be involved in intratumoral metastasis during the process of tumor cell metastasis, but the mechanism has not yet been elucidated.

Intratumoral metastasis to renal cell carcinoma may be suspected when changes are observed in the growth rate of the renal tumor or in the levels of tumor markers for other cancers. In the present case, the FDG-PET findings also changed over time, showing two accumulations within the renal tumor compared with the findings of the previous scan. In patients with renal cell carcinoma in addition to another primary cancer in a different organ, the FDG-PET findings, CT results, and tumor marker levels may aid in the diagnosis of intratumoral metastasis.

Although the tumor diameter was > 7 cm in this case, PN was performed. The surgical difficulty was not considered high because the RENAL score was 5 points (> 50% of the tumor protruded outside the kidney, the distance to the urinary tract or renal sinus was > 7 mm, and the tumor was located above the upper pole) [14]. Furthermore, preoperative CT and FDG-PET showed that the renal tumor might be a metastasis of colon cancer. Considering the need for postoperative adjuvant therapy and the patient’s strong desire to preserve renal function, PN was performed instead of total nephrectomy. Postoperative imaging showed no renal recurrence and the postoperative tumor marker levels declined, suggesting that PN was effective in controlling the renal cancer. In addition, the minimally invasive technique of laparoscopic PN was chosen not only to preserve renal function but also to prepare the patient for postoperative adjuvant therapy and recurring treatment. The use of robotic surgery is expected to become more widespread, further facilitating minimally invasive treatment of renal cancer.

In conclusion, we have herein described a patient who underwent left PN for a renal tumor following colectomy. The patient was then diagnosed with intratumoral metastasis of colon cancer to chromophobe renal cell carcinoma.
